# A Single Dose of Nitrate Increases Resilience Against Acidification Derived From Sugar Fermentation by the Oral Microbiome

**DOI:** 10.3389/fcimb.2021.692883

**Published:** 2021-06-03

**Authors:** Bob T. Rosier, Carlos Palazón, Sandra García-Esteban, Alejandro Artacho, Antonio Galiana, Alex Mira

**Affiliations:** ^1^ Department of Health and Genomics, Center for Advanced Research in Public Health, FISABIO Foundation, Valencia, Spain; ^2^ Department of Microbiology, General University Hospital of Elche, FISABIO Foundation, Alicante, Spain

**Keywords:** nitrate, caries, pH buffering capacity, saliva, *Rothia*, acidification, resilience, oral microbiota

## Abstract

Tooth decay starts with enamel demineralization due to an acidic pH, which arises from sugar fermentation by acidogenic oral bacteria. Previous *in vitro* work has demonstrated that nitrate limits acidification when incubating complex oral communities with sugar for short periods (e.g., 1-5 h), driven by changes in the microbiota metabolism and/or composition. To test whether a single dose of nitrate can reduce acidification derived from sugar fermentation *in vivo*, 12 individuals received a nitrate-rich beetroot supplement, which was compared to a placebo in a blinded crossover setting. Sucrose-rinses were performed at baseline and 2 h after supplement or placebo intake, and the salivary pH, nitrate, nitrite, ammonium and lactate were measured. After nitrate supplement intake, the sucrose-induced salivary pH drop was attenuated when compared with the placebo (p < 0.05). Salivary nitrate negatively correlated with lactate production and positively with ΔpH after sucrose exposure (r= -0.508 and 0.436, respectively, both p < 0.05). Two additional pilot studies were performed to test the effect of sucrose rinses 1 h (n = 6) and 4 h (n = 6) after nitrate supplement intake. In the 4 h study, nitrate intake was compared with water intake and bacterial profiles were analysed using 16S rRNA gene Illumina sequencing and qPCR detection of *Rothia*. Sucrose rinses caused a significant pH drop (p < 0.05), except 1 h and 4 h after nitrate supplement intake. After 4 h of nitrate intake, there was less lactate produced compared to water intake (p < 0.05) and one genus; *Rothia*, increased in abundance. This small but significant increase was confirmed by qPCR (p < 0.05). The relative abundance of *Rothia* and *Neisseria* negatively correlated with lactate production (r = -0.601 and -0.669, respectively) and *Neisseria* positively correlated with pH following sucrose intake (r = 0.669, all p < 0.05). Together, these results show that nitrate can acutely limit acidification when sugars are fermented, which appears to result from lactate usage by nitrate-reducing bacteria. Future studies should assess the longitudinal impact of daily nitrate-rich vegetable or supplement intake on dental health.

## Introduction

When sugars are fermented by the oral microbiota, lactate and other organic acids are produced that can decrease the local pH (Marsh, 2003). The presence of sugars and acidic conditions select for saccharolytic and acid-tolerant microorganisms, increasing the fermentation capacity of the microbial community. This leads to the formation of a vicious circle that can result in the acidification of dental plaque to pH levels at which enamel is demineralized (approximately pH 5.5) (Rosier et al., 2018). When demineralization exceeds remineralization, a caries lesion can develop [reviewed by (Pitts et al., 2017)]. In the early stages, caries can be arrested or remineralized with preventive non-invasive therapy (e.g., changes in dietary practices or fluoride availability) (Frencken et al., 2012; Pitts et al., 2017). However, in the later stages when the microbial activity has cavitated the lesion, the damage is mostly irreversible, and the lesion can only be restored through operative therapy.

According to the Global Burden of Disease 2017, untreated caries in permanent teeth was the most common health condition among those evaluated (GBD, 2017 Disease and Injury Incidence and Prevalence Collaborators, 2018). It is estimated that 2.3 billion people suffer from caries of permanent teeth and 530 million children from caries of primary teeth. As such, the cost of treating caries is expensive and believed to consume 5-10% of healthcare budgets in industrialized countries (Petersen et al., 2005; WHO, 2015).

To reduce the global health and financial burden of caries, preventative care is essential. Two behavioural changes that are recommended to prevent caries are reducing the frequency of fermentable sugar intake and brushing twice daily with fluoridated toothpaste (Frencken et al., 2012). Fluoride increases enamel remineralization and resistance against acidic conditions (Pitts et al., 2017; Rosier et al., 2018), whilst inhibiting bacterial carbohydrate fermentation and thereby limiting acidification (López-López and Mira, 2020). Another preventive approach is using arginine as a prebiotic substrate that oral bacteria convert to ammonia – a weak base that increases the local pH (Liu et al., 2012). Combining arginine and fluoride has been found to be more efficient at preventing plaque acidification in the presence of sugar when compared with fluoride alone (Wolff et al., 2013).

A second potential anti-caries prebiotic is nitrate, but current *in vivo* evidence is limited (Rosier et al., 2018). It is estimated that humans obtain more than 80% of dietary nitrate from vegetables – a food group unequivocally associated with health benefits (Link et al., 2004; Wang et al., 2014; Turati et al., 2015; Lundberg et al., 2018). The salivary glands contain electrogenic sialin 2NO_3_^-^/H^+^ transporters that concentrate plasma nitrate into the saliva (Qu et al., 2016), leading to high salivary nitrate concentrations (100–500 μM during fasting, which is ~10x higher than in plasma, and 5-8 mM after a nitrate-containing meal) (Lundberg and Govoni, 2004; Hezel and Weitzberg, 2015). The human body is incapable of metabolizing nitrate, however, certain oral bacteria can effectively reduce salivary nitrate to nitrite (Schreiber et al., 2010). Some of this nitrite is swallowed and converted into nitric oxide (an antimicrobial and bioactive molecule) by enzymatic and non-enzymatic processes inside the human body (Hezel and Weitzberg, 2015). This process is called the nitrate-nitrite-nitric oxide pathway and is associated with various cardiovascular and metabolic benefits, including blood pressure reduction and antidiabetic effects (Hezel and Weitzberg, 2015; Lundberg et al., 2018).

Along with systemic benefits, nitrate appears to contribute to oral health. Salivary nitrate and the nitrate reduction capacity of the oral microbiota have been shown to negatively correlate with caries incidence (Li et al., 2007). Additionally, physiologically relevant nitrate concentrations (1–8.5 mM) can attenuate a pH drop when sugars are fermented by complex oral communities *in vitro* (Li et al., 2007; Rosier et al., 2020a; Rosier et al., 2020b). In a recent *in vitro* study, nitrate decreased the levels of cariogenic genera and lactic acid production, whilst increasing ammonia (a weak base) production and limiting a pH drop caused by sugar fermentation (Rosier et al., 2020a). These results may be explained by the use of lactate as an electron donor by nitrate-reducing bacteria, the bacterial production of ammonia from nitrite by the DNRA pathway, and/or the bacterial production of antimicrobial nitric oxide through denitrification (Li et al., 2007; Schreiber et al., 2010). However, these mechanisms have not been confirmed *in vivo*.

The aim of our current study was therefore to test whether nitrate limits pH acidification when sugar is consumed and study the potential mechanisms involved in this process *in vivo*. The pH buffering effect of nitrate has been observed as soon as 1 h after incubation *in vitro* (Li et al., 2007). Furthermore, the addition of the reduction product, nitrite, to *ex vivo* dental plaque of children limited acidification after 10 minutes of incubation (Yamamoto et al., 2017). We therefore hypothesized that a single dose of nitrate could acutely limit the oral pH drop that results from sugar consumption.

To test this, 12 individuals without active caries received a nitrate-containing supplement which was compared with a placebo in a crossover blinded setting. The individuals rinsed their mouth with a sucrose rinse before and 2 h after the supplement intake. To test the effect of supplement on acidification, salivary pH levels and concentrations of nitrate, nitrite, ammonium and lactate were measured. To confirm the pH buffering effect and study the time frame in which this process occurs, two additional pilot studies were performed to measure the effects 1 h and 4 h after supplementation and changes in microbiota composition were monitored. With this experimental protocol, we aimed to evaluate whether nitrate may be utilised to reduce oral acidification derived from sugar fermentation.

## Materials and Methods

### Experimental Supplements

**Figure 1:**
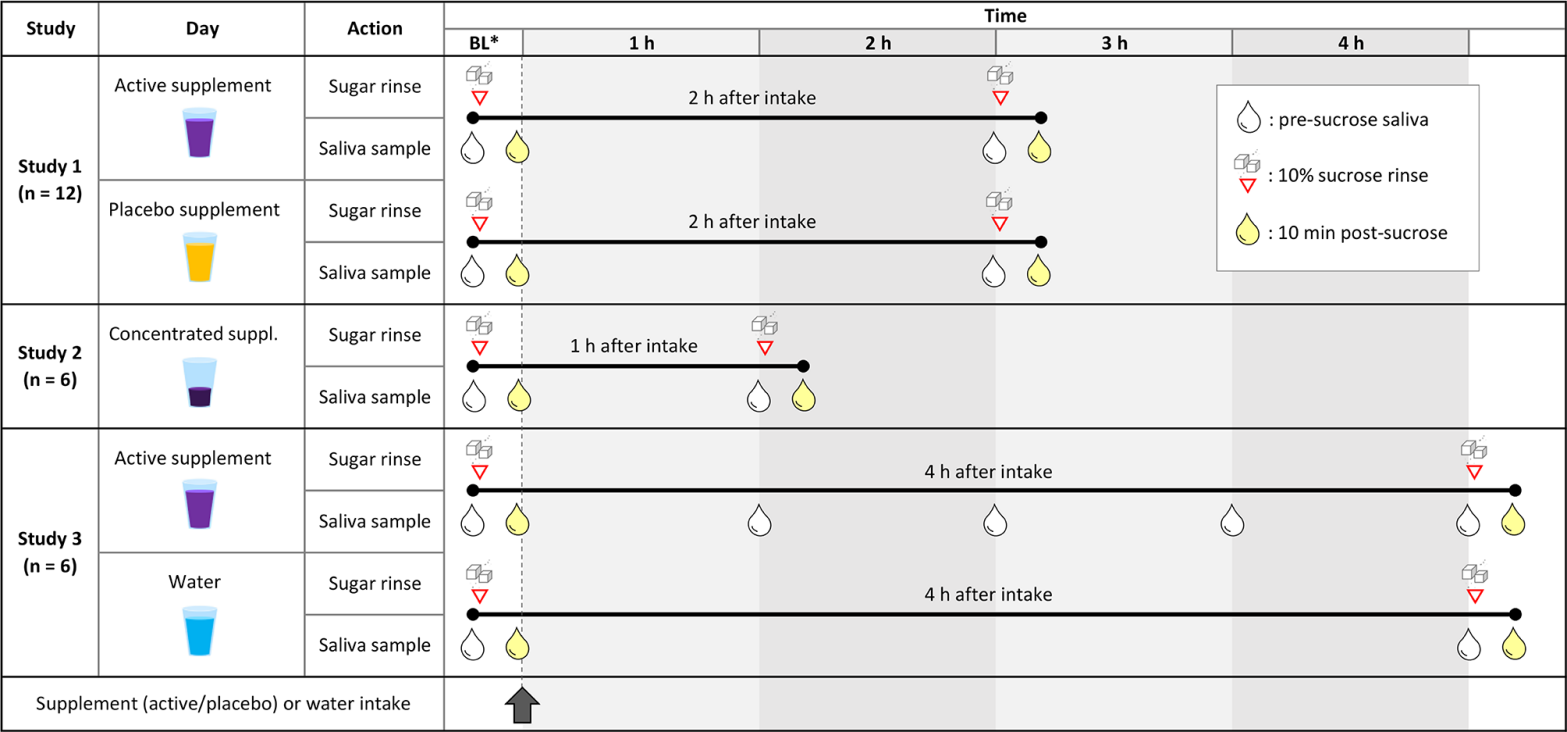
Design of the different clinical studies included in this article. Study 1: Cross-over study design in which 12 individuals received a nitrate-rich supplement (250 mg nitrate in 200 ml) on one day and a nitrate-poor placebo (<6 mg nitrate in 200 ml) on the same day a week earlier (individuals 1-6) or later (individuals 7-12). The effects of both supplements on sucrose rinses were compared. Study 2: Six individuals received a concentrated beetroot extract (300 mg nitrate in 70 ml) to compare the effect of a sucrose rinse before and 1 h after supplement intake. Study 3: Another six individuals received a nitrate rich-supplement (220 mg nitrate in 200 ml) and saliva was taken every hour to monitor changes in physiological parameters. Additionally, the effect of a sucrose rinse after 4 h of supplement intake was compared to a sucrose rinse 4 h after water intake (200 ml) on the previous day. BL, baseline (before supplement intake). Red triangles with sugar cubes show when 10% sucrose rinses were performed, and the drops represent saliva sampling. White drops are saliva samples taken right before a sucrose rinse or without a subsequent sugar, and yellow drops are 10-minute post-sucrose rinse saliva samples.

For the main blinded crossover study (**Figure 1**, study 1), a soluble nitrate-rich supplement powder was used containing dry beetroot extract (*Beta vulgaris*, 3% nitrate), vitamin C and molybdenum (**Supplementary Table 1a**). The supplements were provided by NutriSpain S.A. (Llíria, Valencia, Spain). One 11.5 g dose contained 250 mg nitrate and the recommended daily amounts of Vitamin C (80 mg) and molybdenum (50 µg). This was compared to a placebo (**Supplementary Table 1b**), consisting of the same ingredients, but instead of nitrate-containing beetroot powder, it contained nitrate-poor orange powder (one 11.5 g dose contained <6 mg of nitrate). For the other two pilot studies (**Figure 1**, study 2 and 3), nitrate-containing beetroot extract was used without other ingredients. The powder supplements were weighted to obtain the desired dose of nitrate and dissolved in mineral water (Aguas Cortes) immediately before consumption.

### Study Population

Twelve adults who reported to be systemically healthy were recruited at the FISABIO Institute (Valencia, Spain) to participate in a blinded crossover study (study 1). Individuals could participate with healthy fillings, but were excluded if enamel breakdown or cavitation [i.e., caries with ICDAS scores >2 (Pitts, 2004)] was detected at the moment of sampling, which was visually assessed by an experienced dentist. Other exclusion criteria were the usage of antibiotics in the previous month and weekly usage of mouthwash.

Twelve additional adults were recruited if they reported not to have active caries during their last dental visit. These individuals were divided into two groups of six for the two pilot studies (study 2 and 3).

Unstimulated saliva samples were collected by drooling (Navazesh and Christensen, 1982) in the morning. Individuals were instructed to avoid mouthwash usage in the week(s) of sampling and to have breakfast and brush their teeth with water at least 1 h before arrival on sampling days. For breakfast, they were asked to exclude any vegetable or fruit-derived products, as well as processed meats or other products containing nitrate as a preservative. If measurements were taken on two days, individuals were asked to have the same breakfast twice. All included subjects signed a written informed consent prior to their participation. The study protocol was approved on 2016/05/23 by the Ethical Committee of DGSP-FISABIO (Valencian Health Authority) with the reference BIO2015-68711-R2.

### Sucrose Rinses

Solutions were made of 10% sucrose (Laboratorios Conda, Madrid, Spain) in mineral water. Individuals were instructed to donate 2 ml of saliva (for pre-sucrose measurements), rinse their mouth with sucrose solution for 1 minute, sit down on a chair for 10 minutes, and donate another 2 ml of saliva (for post-sucrose measurements). In all studies, individuals were asked not to consume any food in the period between donating the first and the last saliva samples.

### Study Designs

#### Study 1: Effect of Nitrate-Rich Supplement 2 h After intake Compared to Placebo (Blinded Crossover Design)

Twelve individuals took a nitrate-rich supplement dissolved in mineral water (250 mg nitrate in 200 ml) and a nitrate-poor placebo dissolved in mineral water (<6 mg nitrate in 200 ml) in a blinded crossover setting (**Figure 1**, study 1). Specifically, 6 individuals took the active supplement on one day and the placebo on the same day a week later, while the other 6 individuals took the supplement and placebo in the reversed order. The two vegetable extracts had a different colour, but participants were unaware of the active ingredient of interest (nitrate). On each day, a 10% sucrose rinse was performed at baseline (before supplement intake) and 2 h after supplement intake.

#### Study 2: Effect of Nitrate-Rich Supplement 1 h After Intake Compared To Baseline

In a first pilot study including six individuals, the immediate effect of a concentrated beetroot extract (300 mg nitrate in 70 ml) was tested 1 h after intake (**Figure 1**, study 2). A sucrose rinse was performed before (baseline) and 1 h after supplement intake.

#### Study 3: Effect of Nitrate-Rich Supplement 4 h After intake Compared To Water Intake

In a second pilot study including another six individuals, the effect of a beetroot extract (220 mg nitrate in 200 ml) was tested for 4 h after intake (**Figure 1**, study 3). A sucrose rinse was performed at baseline and 4 h after supplement intake. Additionally, 1 ml saliva was collected at 1 h, 2 h and 3 h after supplement intake to monitor physiological parameters over time. As a control, the same 6 individuals consumed 200 ml of mineral water on another day and sugar rinses were performed at baseline and 4 h after water intake.

### Physiological Measurements in Saliva

Salivary concentrations of nitrate and nitrite, and pH levels were measured with a Reflectoquant reflectometer (Merck Millipore, Burlington, Massachusetts, US) as described by Rosier et al. (2020a). The concentration of ammonium in saliva was measured spectrophotometrically by the Nessler Method (Santarpia et al., 2014). The concentration of lactate in saliva was measured with the Lactate Colorimetric/Fluorometric Assay Kit (BioVision, Milpitas, California, US) following the manufacturer’s instructions. Accuracy of all procedures was confirmed by using standard solutions with known concentrations of the different compounds.

### DNA Isolation

DNA was isolated from 24 saliva samples of study 3, corresponding to the baseline and 4 h pre-sucrose samples of both days (supplement and water intake). To obtain a bacterial pellet, 250 µl were centrifuged and the supernatant was removed. The pellet was dissolved in 100 µl PBS and DNA was extracted from the samples using the MagNA Pure LC DNA isolation kit (Roche Diagnostics, Mannheim, Germany) with the addition of a chemical lysis step with an enzymatic cocktail containing lysozyme, mutanolysin and lysostaphin, following Dzidic et al. (2018) and Rosier et al. (2020a). DNA concentrations were measured using a QubitTM 3 Fluorometer (Thermofisher, Loughborough, UK).

### 16S rRNA Gene Illumina Sequencing

An Illumina amplicon library was prepared following the 16S rRNA gene Metagenomic Sequencing library preparation Illumina protocol (Part #15044223 Rev. A). The primer sequences used in this protocol were; 16S Amplicon 341F (TCGTCGGCAGCGTCAGATGTGTATAAGAGACAGCCTACGGGNGGCWGCAG) and 805R (GTCTCGTGGGCTCGGAGATGTGTATAAGAGACAGGACTACHVGGGTATCTAATCC), which amplify the V3-V4 hypervariable regions of the gene. Following 16S rDNA gene amplification, DNA was sequenced with an Illumina MiSeq Sequencer according to manufacturer’s instructions using the 2x300 bp paired-ends protocol.

### Taxonomic Classification Using DADA2

To process the paired-end fastq files, an amplicon sequence variant (ASV) table was obtained using the DADA2 pipeline (v1.8) in R (Callahan et al., 2016). In short, the forward and reverse reads were trimmed, removing the v5-v6 primer sequences and low-quality bases at the end of reads. Reads with any ambiguous N base or exceeding 5 expected errors were also discarded. The forward and reverse pairs were merged together, with a minimum overlap of 12 bases and a maximum mismatch of 1 base in the overlapping region, to obtain the single denoised variants. After chimeric variant removal, the final amplicon sequence variants (ASVs) were mapped onto the Homo sapiens genome (assembly GRCh38.p13), using Bowtie2 (Langmead and Salzberg, 2012) (v2.3.5.1), in order to remove reads from the host. The Silva database (Quast et al., 2013; Yilmaz et al., 2014) (v138) was set as reference to assign taxonomy to each ASV. Genus classification was achieved using the DADA2 naive Bayesian classifier method. The ASVs with an assigned genus but without exact species, were aligned using the Blastn tool (Altschul et al., 1990) (v2.10.0+) against the Silva database with a minimum of 97% of identity.

### qPCR of *Rothia*

The total amount of *Rothia* cells in saliva were analysed through quantitative PCR (qPCR) amplification of the *Rothia* nitrate reductase *narG* gene. Primers sequences were designed to be specific for the *Rothia* genus, using conserved regions of *narG* from *Rothia mucilaginosa*, *R. dentocariosa* and *R. aeria*. The forward sequence was 5’-ACA CCA TYA AGT ACT ACGG-3’ and the reverse 5’-TAC CAG TCG TAG AAG CTG-3’. Reactions of 20 μl were added per well of a qPCR plate, consisting of 10 μl of Light Cycler 480 SYBR Green I Master mix (Roche Life Science, Penzberg, Germany), 0.4 μl of each specific primer (10 μM), 6.7 μl water and 2.5 μl of template DNA (DNA isolated from saliva and pre-diluted to 2 ng/l). Each sample was added in duplicate and measurements were performed using a Light Cycler 480 Real-Time PCR System (Roche Life Science) with the following conditions: 95°C for 2 min, and 40 cycles of 95°C for 30 s, 60°C for 20 s, and 72°C for 25 s. Negative controls were added, as well as a standard curve, consisting of a series dilution of an equimolar DNA mix of three *Rothia* species (*R. mucilaginosa* DSM-20746, *R. dentocariosa* DSM- 43762, *R. aeria* DSM-14556) quantified with a QubitTM 3 Fluorometer (Thermofisher). Based on genome sizes, the cell number was calculated, assuming a single copy of the *narG* gene per cell.

### Data Analysis

Physiological parameters and qPCR data at different time points were compared with SPSS (v27) using Wilcoxon sign rank tests. Correlations between physiological parameters were explored using Spearman-rho in SPSS.

The salivary microbiota composition of six individuals was determined before and 4 h after nitrate intake. For analysis of the bacterial composition, R programming language (R Core Team, 2014) was used as described by Johnston et al. (2021). In short, Wilcoxon sign rank (wilcox.test function) and Spearman’s rho (cor.test function) tests were performed. Additionally, the Vegan library of R (Oksanen et al., 2017) was used for Adonis tests (Permutational Multivariate Analysis of Variance Using Distance Matrices) and the visualization of bacterial composition in a two-dimensional map using constrained correspondence analysis (CCA). For these analyses, a species or genus was included if it was present in 70% of the samples from at least one of the two groups (relevant for Wilcoxon tests) or from the given group under study (relevant for Spearman’s correlations) with an abundance superior to ten times the smallest percentage above zero. Only cases of genera with a median abundance >0.1% were discussed. Data were visualized with GraphPad PRISM (v9).

## Results

### pH-Buffering Effect 2 h After Nitrate Intake Compared to Placebo

**Figure 2:**
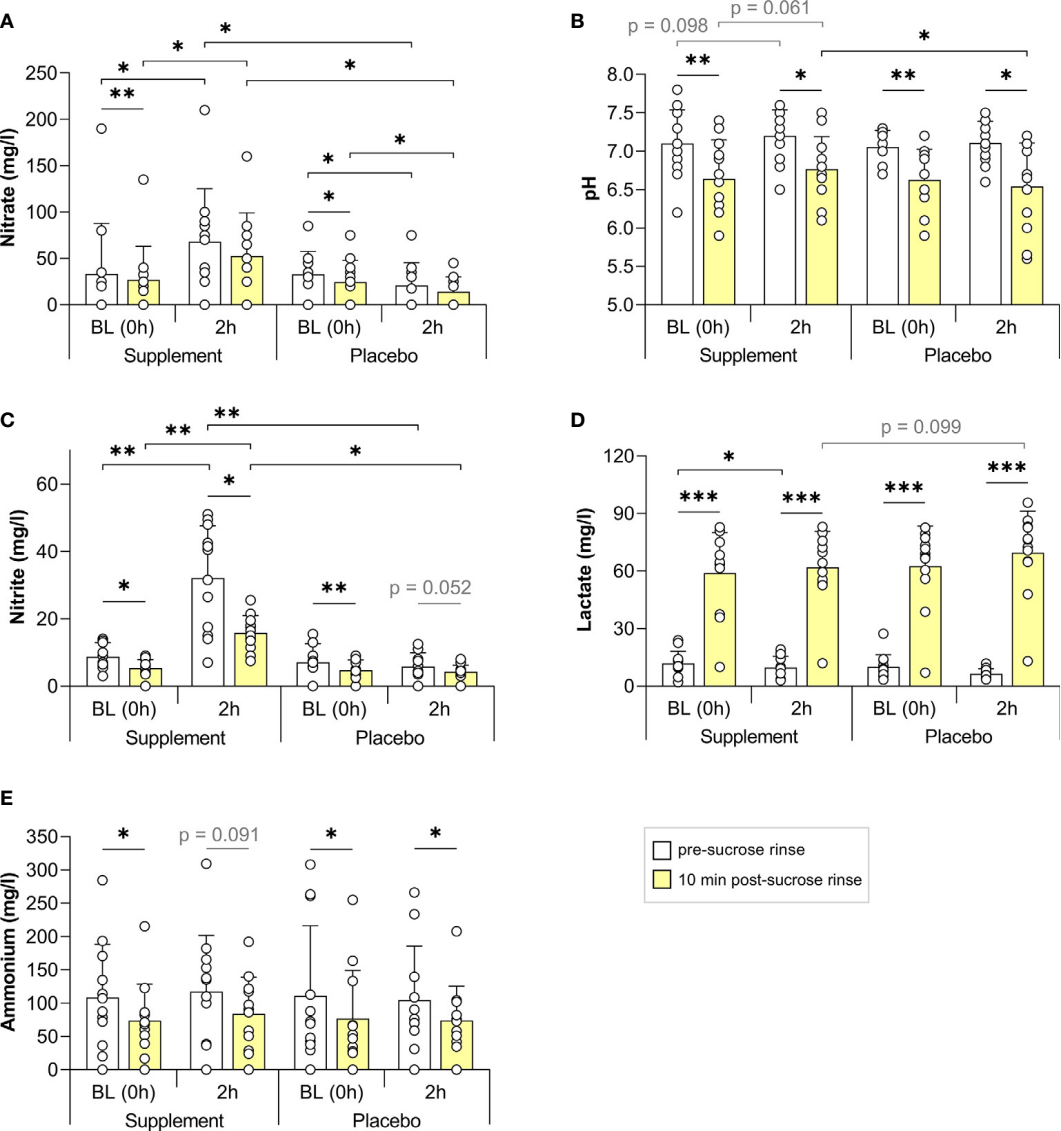
The effect of a nitrate-rich supplement on sucrose rinses 2 h after intake compared with a placebo. In 12 individuals, salivary nitrate **(A)**, pH **(B)**, nitrite **(C)**, lactate **(D)** and ammonium **(E)** were measured. Sugar rinses were performed at baseline (BL, 0 h) on the two different days (supplement day and placebo day) and 2 h after intake of a nitrate-rich supplement (250 mg nitrate in 250 ml) or nitrate-poor placebo (<6 mg nitrate in 250 ml). Saliva samples were collected immediately prior to the sugar rinse (pre-sucrose, white bars) and 10 min after the sugar rinse (post-sucrose, yellow bars). The bars and small white circles represent the averages and individual donor’s data, respectively. Wilcoxon tests were used to compare the pre-sucrose with the post-sucrose measurements. Additionally, the BL pre- and post-sucrose measurement were compared with 2 h pre- and post-sucrose measurement, respectively. Finally, every measurement on the supplement day was compared with the same measurement on the placebo day. Significant changes (*p < 0.05, **p < 0.01, ***p < 0.005) and trends (grey text, p = 0.05 - 0.1) are shown.

In the first study, including twelve individuals (5 males and 7 females, age 25-60), the consumption of a nitrate-rich beetroot supplement dissolved in mineral water (250 mg nitrate in 200 ml) significantly increased salivary nitrate and nitrite after 2 h (p < 0.05, **Figures 2A, C**, or see **Supplementary Spreadsheet** for all physiological parameters and participants’ information). In contrast, 2 h after the placebo supplement dissolved in mineral water (<3 mg nitrate in 200 ml), nitrate levels had decreased significantly, whilst nitrite levels did not vary. Following sucrose rinses, the pH dropped significantly in all cases (**Figure 2B**). However, on average, the pH dropped 0.23 points less when using the nitrate-rich supplement compared with the placebo (p < 0.05).

There was also a trend for lower lactate concentrations 2 h after the nitrate supplement compared with the placebo (**Figure 2D**, p = 0.099). Furthermore, there was a trend for higher salivary pH 2 h after nitrate supplement intake compared with baseline, both in the pre-sucrose rinse pH (p = 0.098), and after the sucrose rinse (p = 0.061). These trends were not found when taking the placebo (p = 0.439 and p = 0.339, respectively).

Nitrite levels dropped after all sucrose rinses (p < 0.05 on the supplement day and p = 0.052 on the placebo day), indicating possible metabolization of this compound or dilution by rinsing, whilst nitrate only dropped after sucrose at the baseline rinses (p < 0.05). Lactate increased after all sucrose rinses (p < 0.005), whereas ammonium dropped significantly after all sucrose rinses (p < 0.05), except 2 h after nitrate supplement intake (p = 0.091, **Figure 2E**).

**Figure 3:**
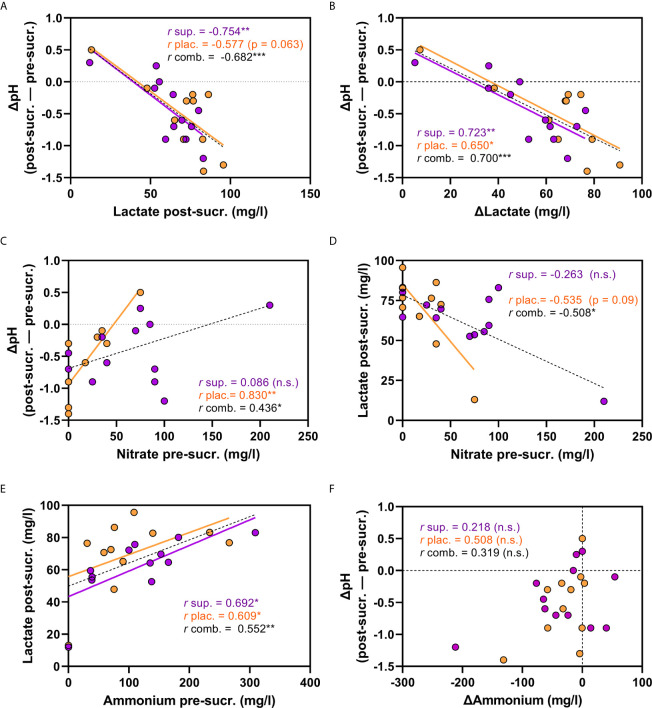
Correlations between physiological parameters 2 h after nitrate-rich supplement and placebo intake. In **(A–F)**, correlations between different physiological parameters are shown. The purple dots are measurements 2 h after nitrate-rich supplement intake (n = 12) and the orange dots 2 h after placebo intake on a different day (n = 12). The black dotted lines are the linear regression curves when combining the measurements after supplement and placebo intake (total n = 24). The ΔpH is the pH difference between the pre-sucrose measurement and the post-sucrose measurement (negative values are a pH drop). **(A)** ΔpH and lactate detected post-sucrose. **(B)** ΔpH and Δlactate. **(C)** ΔpH and salivary nitrate (pre-sucrose) **(D)** lactate detected post-sucrose and salivary nitrate (pre-sucrose). **(E)** salivary ammonium (pre-sucrose) and lactate detected post-sucrose. **(F)** ΔpH and Δammonium. sup., supplement; plac., placebo; comb., combined; pre-sucr., pre-sucrose measurements; post-sucr., 10 min post-sucrose measurement. Spearman-Rho correlations (*r*) were calculated 2 h after supplement (sup.) or placebo (plac.) intake, or both combined (comb.). P-values and linear regression curves are shown if trends were found (p 0.05-0.1). *p < 0.05, **p <0.01, ***p <0.001, n.s, not significant. Δ = post-measurement – pre-measurement.

The change in pH after the sugar rinse (ΔpH) at 2 h negatively correlated with the lactate detected post-sucrose (**Figure 3A**) and with the change in lactate after the sugar rinse (Δlactate, **Figure 3B**), indicating that lactate production is a good proxy for the magnitude of the pH drop. It is therefore interesting that the salivary nitrate (pre-sucrose) correlated negatively with the lactate produced (r = -0.508, p < 0.5, **Figure 3D**). In line with these results, the nitrate levels showed a significant positive correlation with the ΔpH (r = 0.436, p < 0.05, **Figure 3C**), indicating that nitrate availability at 2 h was associated with lower pH drops. No evidence of pH buffering by ammonium production was obtained as changes in ammonium after the sugar rinse (Δammonium) did not correlate significantly with the ΔpH (**Figure 3F**). An unexpected positive correlation was found between salivary ammonium (pre-sucrose) and the lactate detected after sugar rinsing (r = 0.552, p < 0.01, **Figure 3E**). Similar correlations were observed when analysing the baseline measurements together with measurements after supplement intake of all individuals that participated in the three studies of this manuscript (**Supplementary Figure 1**).

### Immediate pH-Buffering Effect 1 h After Nitrate Intake

**Figure 4:**
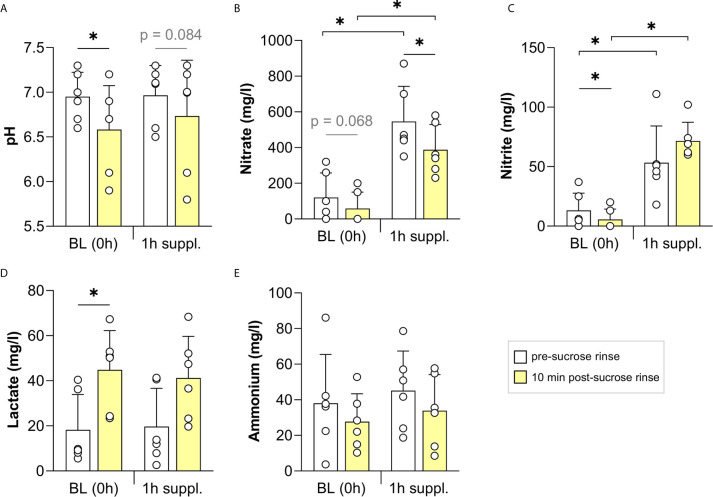
The effect of a concentrated nitrate-rich supplement on sucrose rinses 1 h after intake. In six individuals, the salivary pH **(A)** and concentrations of nitrate **(B)**, nitrite **(C)**, lactate **(D)** and ammonium **(E)** were measured. Sugar rinses were performed at baseline (BL, 0 h) and 1 h after intake of a nitrate-rich supplement (300 mg nitrate in 70 ml). Saliva samples were collected immediately prior to the sugar rinse (pre-sucrose, white bars) and 10 min after the sugar rinse (post-sucrose, yellow bars). The bars and small white circles represent the averages and individual donor data, respectively. Wilcoxon tests were used to compare the pre-sucrose with the post-sucrose measurements. Additionally, the BL pre- and post-sucrose measurement were compared with 2 h pre- and post-sucrose measurements, respectively. Significant changes (*p < 0.05) and trends (grey text, p = 0.05 - 0.1) are shown.

In the second study, including six individuals (3 males and 3 females, age 24-46), the potential effect of a more concentrated nitrate supplement (300 mg nitrate in 70 ml) was tested only 1 h after intake (**Figure 4**). The sucrose rinses led to an average pH drop of 0.37 points at baseline, which was significant (p < 0.05, **Figure 4A**), and 0.23 points after supplement intake, which showed a trend towards significance (p = 0.084). However, there was some heterogeneity in this outcome: in 4 out of 6 participants, the nitrate supplement limited or fully prevented the pH drop caused by the sucrose rinse and in the other two participants, the pH drop was as strong as at baseline (**Supplementary Spreadsheet**). Lactate also increased significantly after the sucrose rinse at baseline (p < 0.05), but not 1 h after taking the supplement (**Figure 4D**). Ammonium levels dropped after the sugar rinses, but the difference was not significant (**Figure 4E**). When combining the data of the three studies in this manuscript, significant drops in nitrate, nitrite and ammonium after sucrose rinses were confirmed (p < 0.05, **Supplementary Figure 3**).

### pH-Buffering Effect 4 h After Nitrate Intake Compared to Water Intake

**Figure 5:**
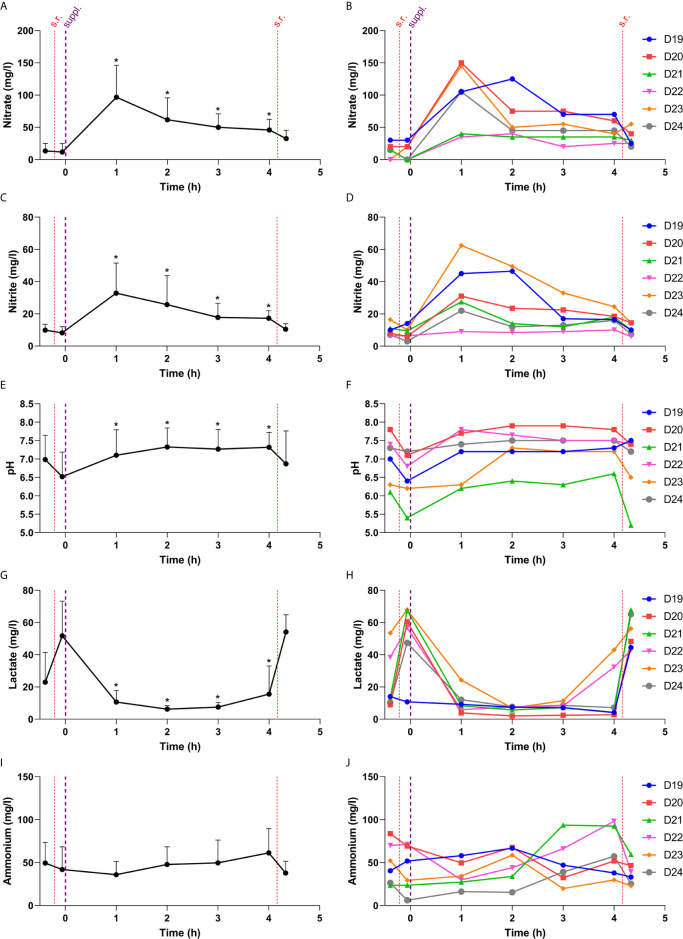
Changes in physiological parameters during the 4 h after taking a nitrate-rich supplement. In this figure, the salivary nitrate **(A, B)**, nitrite **(C, D)**, pH **(E, F)**, lactate **(G, H)** and ammonium **(I, J)** are shown over time. The red lines are sugar rinses dividing pre- and post-sucrose measurements at baseline (0 h) and after 4 h of supplement intake. The thicker purple line represents when the nitrate-rich supplement was consumed. On the left side, the averages are shown with standard deviations. The time points 1 h, 2 h, 3 h and 4 h (pre-sucrose) were compared to 0 h (pre-sucrose). * = compared to 0 h (pre-sucrose), the p-value was < 0.05. On the right side, the individual donors are shown. S.r., sugar rinse; Suppl., supplement intake. In Figure 6, the pre- and post-sucrose measurements are compared.

In the third study, including another six individuals (3 males, 3 females, age 25-33), the effect of a nitrate supplement (220 mg nitrate in 200 ml) on pH and the salivary microbiota composition was tested 4 h after intake and this was compared with water intake. On the day of nitrate intake, saliva samples were collected every hour for physiological measurements, including nitrate, nitrite, pH, lactate and ammonium (**Figure 5**). After supplement intake, salivary nitrate and nitrite levels peaked at 1 h and stayed elevated over the entire study period of 4 h (**Figures 5A, B**, p < 0.05). In contrast, lactate levels dropped and stayed lower over the entire period compared with the pre-sucrose baseline measurement (**Figure 5G**). The pH was higher at all time points after supplement intake (p < 0.05 when comparing 0 h with 1-4 h, **Figure 5E**).

**Figure 6:**
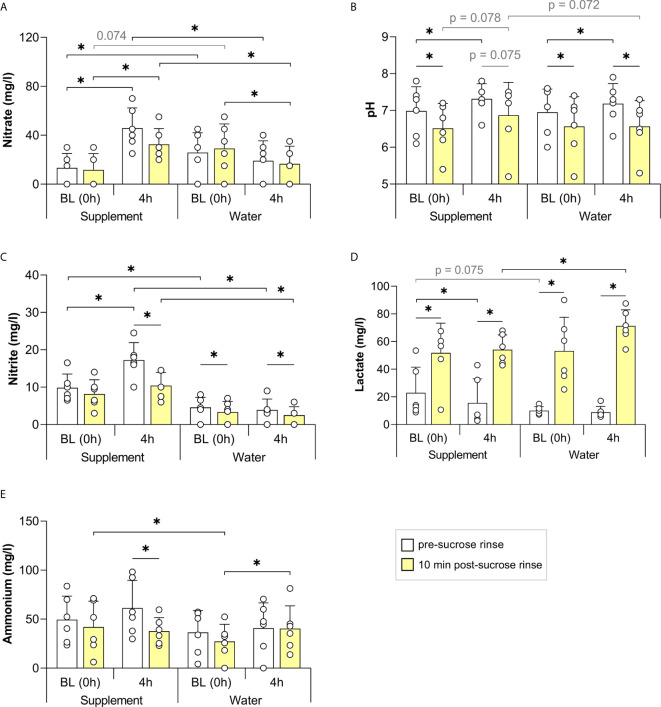
The effect of a nitrate-rich supplement on sucrose rinses 4 h after intake compared with water. In 6 individuals, salivary nitrate **(A)**, pH **(B)**, nitrite **(C)**, lactate **(D)** and ammonium **(E)** were measured. Sugar rinses were performed at baseline (BL, 0 h) of the two different days (supplement day and water day) and 2 h after intake of a nitrate-rich supplement (220 mg nitrate in 200 ml) or water (200 ml). Saliva samples were collected immediately prior to the sugar rinse (pre-sucrose, white bars) and 10 min after the sugar rinse (post-sucrose, yellow bars). The bars and small white circles represent the averages and individual data, respectively. Wilcoxon tests were used to compare the pre-sucrose with the post-sucrose measurements. Additionally, the BL pre- and post-sucrose measurements were compared with 2 h pre- and post-sucrose measurements, respectively. Finally, every measurement of the supplement day was compared with the same measurement on the water day. Significant changes (*p < 0.05) and trends (grey text, p = 0.05 - 0.1) are shown.

The salivary pH dropped significantly after a sucrose rinse at baseline and also 4 h after water intake (**Figure 6B**), and a trend (p = 0.075) towards a pH decrease was observed 4 h after the nitrate-rich supplement intake. Four hours after nitrate supplement ingestion, there was also a trend (p = 0.072) towards the post-sucrose pH being less acidic, whereas lactate production was significantly lower (p < 0.05), compared with water intake.

Regardless of supplement or water intake, the pre-sucrose salivary pH increased after 4 h (both p < 0.05, **Figure 6**), which may be due to the natural buffering effect of saliva over time after breakfast. Specifically, 4 h after supplement intake, the pH increased from an average of 6.98 (SD 0.66) to 7.32 (SD 0.41), whilst 4 h after water intake, it increased from 6.95 (SD 0.63) to 7.18 (SD 0.55).

### Changes in Microbiota Composition 4 h After Nitrate Intake

**Figure 7:**
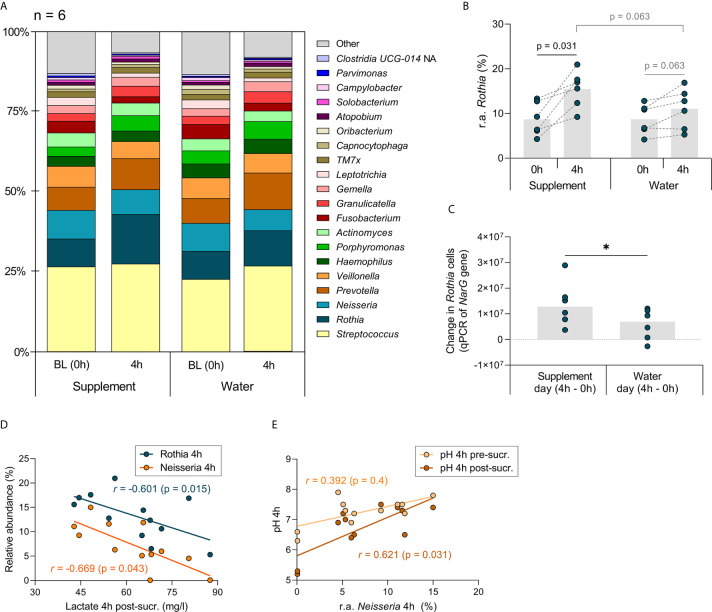
Genus level analysis of study 3 (comparing a nitrate-rich supplement intake with water intake). In **(A)**, the relative abundance of the genera detected in the 6 individuals of study 3 are represented in bar charts. Based on the median, the 20 most abundant genera are shown. The grey boxes with “other” contain genera < 0.34% abundance grouped together. In **(B)**, the relative abundance (r.a.) of the genus *Rothia* is shown, which showed the strongest trend towards an increase when comparing BL (0 h) with 4 h after nitrate-rich supplement intake (p value = 0.031, adjusted p-value = n.s.). The grey bars represent the averages and the dark cyan dots the data from individual participants. The higher increase of *Rothia* after taking the supplement compared with water intake was confirmed by qPCR in **(C)** the increase of *Rothia* cells from 0 h to 4 h, based on the number of copies of the *Rothia* nitrate reductase *narG* gene, was higher after taking the supplement than after taking water (*p < 0.05). **(D)** the measurements 4 h after water intake and 4 h after supplement intake were combined (n = 12) and a lactate post-sucrose correlated negatively with the abundance of *Rothia* and *Neisseria*. In **(E)**, the correlation with *Neisseria* between pH pre-sucrose and pH post-sucrose is shown. Because of the small sample size, unadjusted p-values are shown.

Preliminary data on bacterial composition changes was obtained by comparing the salivary communities of six individuals before (0 h) and 4 h after nitrate supplement or water intake (**Figure 7**). The general bacterial community structure was not affected significantly by the nitrate supplement compared to water intake (no significant changes based on Adonis and CCA p-values, Supplementary **Figure 2**). There was a trend towards *Rothia* being elevated 4 h after nitrate supplement intake compared with water intake (p = 0.063, **Figure 7B**). When comparing 0 h and 4 h (p < 0.05), *Rothia* increased significantly after supplement intake and showed a trend of increase following water intake (p = 0.063). A larger increase in *Rothia* cells after nitrate supplement intake was confirmed by qPCR (p < 0.05, **Figure 7C**).

On a species taxonomic level (**Supplementary Figure 4A**), *R. dentocariosa*, increased significantly from 0 h to 4 h after nitrate supplement intake (p < 0.05), but not after water intake (**Supplementary Figure 4C**). Another *Rothia* species, *R. mucilaginosa* increased significantly from 0 h to 4 h after both nitrate supplementation and water intake (both p < 0.05, **Supplementary Figure 4B**). However, there was a trend of *R. mucilaginosa* being more abundant 4 h after supplementation compared with water intake (p = 0.063), whilst two low abundance species (median abundance <0.15%); *Prevotella salivae* and *Corynebacterium durum*, were significantly less abundant (p < 0.05, **Supplementary Figure 4D, 4E**).

When combining the measurements from all samples collected 4 h after supplement and water intake, the relative abundance of the *Rothia* and *Neisseria* negatively correlated with lactate production post-sucrose (r = -0.601 and -0.669, respectively, both p < 0.05, **Figure 7D**). *Neisseria* also positively correlated with the post-sucrose pH (r= 0.621, p < 0.05), but not with the pre-sucrose pH (**Figure 7E**). *Peptostreptococcus*, which had a median abundance of only 0.22% after 4 h of supplement and water intake, positively correlated with post-sucrose lactate production (r = 0.734, p < 0.01) and negatively correlated with post-sucrose pH (r = -0.825, p < 0.001, **Supplementary Spreadsheet**).

## Discussion

Our data show that a single dose of nitrate around the acceptable daily intake (i.e., 3.7 mg/kg, which would imply 222-296 mg for an adult of 60-80 kg) can attenuate acidification following sugar fermentation by the oral microbiota in individuals with good dental health (without enamel breakdown or untreated cavities). The different studies presented in the current manuscript indicate that increased protection against sucrose can happen as early as 1 h, and last for at least 4 h after nitrate-rich beetroot supplementation. We identify that, in this timeframe, the main pH buffering mechanism after sucrose rinsing was lactate usage. Preliminary evidence was also obtained suggesting that the bacteria involved in pH buffering are *Neisseria* and *Rothia* – known nitrate reducers – as their abundance in saliva was negatively associated with lactate production after sucrose rinsing. *Rothia* appears to react quickly to nitrate, increasing in number 4 h after nitrate intake, whereas *Neisseria* may act at a transcriptomic level.

In total, 24 healthy individuals participated in the three studies, performing mouth rinses with a 10% sucrose solution at baseline (0 h) and 1 h (n = 6), 2 h (n = 12) or 4 h (n = 6) after nitrate-rich beetroot supplementation. Immediately prior to sucrose rinsing (pre-sucrose) and 10 minutes after (post-sucrose), salivary pH was measured to determine the sucrose-induced pH drop (ΔpH). In all samples, the lactate produced post-sucrose showed a strong negative correlation (p < 0.0001) with the ΔpH, confirming its role in acidification after sugar rinsing (Matsui and Cvitkovitch, 2010).

In the 12 individuals for which pH was monitored 2 h after nitrate intake, the effect of the sucrose rinse was compared to a sucrose rinse 2 h after placebo intake on another day (blinded cross-sectional design). After nitrate rich-supplementation, the post-sucrose pH drop was significantly lower (p < 0.05), and there was a trend towards less lactate production post-sucrose (p = 0.099), compared with placebo intake. Specifically, the pH drop was limited by 0.23 points on average, which could prevent or reduce the time during which enamel is exposed to critical, demineralizing pH levels (around pH 5.5) in some individuals. In our study, excluding caries active patients, only 3 out of 84 sucrose rinses caused a salivary pH decrease below pH 5.5. Therefore, ensuing studies would benefit from studying these effects in a population with active caries, as well as focusing on plaque pH, which is expected to undergo a larger pH drop. In the individuals without active caries in our current manuscript, salivary nitrate correlated negatively with lactate production and positively with the ΔpH (the more nitrate, the smaller the pH drop) (both p < 0.05). This data supports previous *in vitro* studies showing that nitrate (at concentrations found in saliva) prevented or limited a pH drop when incubating oral communities with sugar or sugar-rich medium during periods of 1 to 9 h (Li et al., 2007; Rosier et al., 2020a; Rosier et al., 2020b).

In the six individuals monitored for 4 h after nitrate intake, the effect of the sucrose rinse was compared with a sucrose rinse 4 h after water intake on the previous day. Compared with water intake, there was significantly less lactate produced post-sucrose (p < 0.05), and a trend was observed for a lower post-sucrose pH drop (p = 0.072) after nitrate intake. Lactate is the ion of lactic acid, which is the main organic acid involved in caries development (Pitts et al., 2017). A decrease of lactate can result from nitrate-reducing bacteria which use lactate as an electron donor and carbon source during nitrate reduction (Wicaksono et al., 2021). The negative correlation between salivary nitrate pre-sucrose and the lactate produced post-sucrose in this study (p < 0.05) suggests that lactate was used in the presence of nitrate. In a previous *in vitro* study, we incubated saliva in sugar-rich medium and observed that nitrate (6.5 mM = 403 mg/l) decreased the lactate production and prevented a pH drop after 5h and 9h of incubation (Rosier et al., 2020a). In another recent clinical study, the use of chlorhexidine mouthwash impaired nitrate-reduction, whilst increasing lactate production and decreasing the pH buffering capacity of saliva (Bescos et al., 2020). This indicates that nitrate reduction is an important regulator of lactate levels in the oral cavity, a hypothesis which fits with environmental microbiology studies, where nitrate reduction has also found to increase lactate usage (Tiedje, 1988; Li et al., 2007; Koopman et al., 2016). However, it cannot be excluded that (in parallel) lactate production is decreased (e.g., by inhibition of lactate-producing bacteria or enzymes) and this possibility should be tested in future *in vitro* studies.

In our previous *in vitro* study, we also observed an increase in *Rothia* and *Neisseria* 5 h after incubating saliva with 6.5 mM nitrate (Rosier et al., 2020a). Four hours after nitrate intake in our current manuscript, a significantly larger increase in *Rothia* cells (measured with qPCR) was observed when compared with water intake (p < 0.05). Compositional data, determined by 16S rRNA gene Illumina sequencing in the samples of six individuals, showed similar trends: an increase in the genus *Rothia* and the species *R. mucilaginosa* and *R. dentocariosa* after nitrate intake. However, only moderate *Rothia* increases were detected after 4 h and therefore we expect that an important fraction of this immediate pH buffering mechanism is due to transcriptomic changes. Previous clinical studies have found higher levels of *Rothia* and *Neisseria* after 10 days (Vanhatalo et al., 2018), and *Rothia mucilaginosa* and *Neisseria flavescens* after 6 weeks (Velmurugan et al., 2016), of daily nitrate-rich beetroot juice intake. Additionally, Vanhatalo and colleagues found that after 10 days, *Veillonella* and *Prevotella* had decreased (Vanhatalo et al., 2018). In our study, we did not find an increase in *Neisseria*, nor decreases in any genera. Only two species, *Prevotella salivae* and *Corynebacterium durum*, appeared to be less abundant in saliva 4 h after nitrate intake compared with water intake. Thus, preliminary composition data of our study suggest that an initial increase in *Rothia* can happen as soon as 4 h after intake of the first nitrate dose, whereas *Neisseria* and other taxonomic changes may require more time to develop in the presence of nitrate. To better understand the response to sugar in the presence of nitrate, future clinical studies should focus on metagenomic and metatranscriptomic changes directly measured in dental plaque. Additionally, the accumulative effect of nitrate should be determined when taking daily doses over several days. Regarding this, pH buffering effects were observed after 2-4 weeks of daily toothbrushing with an arginine-containing dentifrice (Santarpia et al., 2014), suggesting that continuous exposure to arginine induced an ecological shift in plaque bacterial populations, and future studies should address this possibility in relation to nitrate exposure and oral health parameters.

The relative abundance of *Rothia* and *Neisseria* in saliva negatively correlated with the lactate produced after sugar rinsing. Additionally, the levels of *Neisseria* positively correlated with the post-sucrose pH, but not with the pre-sucrose pH. This indicates that *Rothia* and *Neisseria* may be involved in the nitrate-mediated pH buffering mechanism observed throughout the current study. We recently showed that oral communities to which a *Rothia* isolate (*R. mucilaginosa* or *R. aeria*) was added produced less lactate in the presence of nitrate *in vitro* (Rosier et al., 2020b). This nitrate-dependent difference was not found in the same oral communities without *Rothia*, indicating that the added *Rothia* consumed lactate in the presence of nitrate. Likewise, Wicaksono and colleagues found that nitrate-reduction by *Veillonella* atypica or *Veillonella parvula* was linked to lactate usage (Wicaksono et al., 2021). Interestingly, both *Rothia* and *Neisseria* have been associated with oral disease-free individuals [previously discussed in Rosier et al., 2018; Rosier et al., 2020a; Rosier et al., 2020b)], including caries-free individuals (Crielaard et al., 2011; Alcaraz et al., 2012; Belda-Ferre, 2012), and their ability to use lactate may be linked with dental health.

In a recent and pioneering clinical study, Burleigh et al. (2020) showed that a single high dose of nitrate (770 mg) in the form of concentrated beetroot juice limited acidification 5-15 minutes after intake of a citric acid- and sugar-rich sport drink, compared to a nitrate-depleted beetroot juice (Burleigh et al., 2020). In their study, the acidification induced by the sport drink was likely a combination of the effects of citric acid and sugar. The sucrose rinses in our study consisted of mineral water with 10% sucrose, confirming that nitrate limited pH drops caused by sucrose fermentation. In our pilot experiment with six individuals, we showed that whilst the pH dropped significantly by 0.37 points after sucrose rinsing (p < 0.05), 1 h after a nitrate rich supplement (300 mg nitrate) intake, the pH decrease reached only 0.23 points after sucrose rinsing (p = 0.084). Similarly, the post-sucrose lactate significantly increased prior to nitrate-rich supplement intake (p < 0.05), but did not 1 h after intake (p = 0.116). This indicates that immediately after the supplement intake, nitrate can induce resilience against lactate production when sugar is consumed, limiting a pH drop in some individuals.

Along with lactate consumption, further reduction of nitrite to ammonia may contribute to the pH buffering effect of nitrate (Li et al., 2007; Schreiber et al., 2010). This idea is supported by our previous *in vitro* data, showing elevated ammonia production after 5h and 9h by oral biofilm communities in the presence of nitrate (Rosier et al., 2020a). In the current manuscript, we did not find a significant increase of salivary ammonia in any of the clinical studies. In all participants, the ammonium detected at baseline was 75.99 mg/l, while 1-4 h after nitrate supplement intake, it was 85.23 mg/l, but this difference was not significant (p = 0.114, **Supplementary Figure 3E**). Additionally, 10 minutes after sugar rinsing, we did not detect an increase in salivary ammonium in individuals who consumed a nitrate-rich supplement. Thus, in these *in vivo* conditions, ammonia production did not appear to be a main pH buffering mechanism in saliva. It is possible that ammonia is produced and accumulates in dental plaque as DNRA is usually a strictly anaerobic process (Schreiber et al., 2010), but may be diluted in saliva by salivary clearance and swallowing. Additionally, ammonia production may be an important buffering mechanism in saliva with different amounts and/or frequencies of nitrate intake. For example, Burleigh and colleagues (2019) observed an increase in salivary pH after 7 days of daily nitrate-rich beetroot juice supplementation. In future clinical experiments, ammonia should be measured after long-term (chronic) nitrate consumption to elucidate its role in salivary pH elevation.

Instead of finding a significant increase in ammonium after nitrate consumption as hypothesized, an unexpected strong correlation was found between ammonium in saliva (pre-sucrose) and lactate produced after sucrose. This correlation was strong and consistent in different samples (p < 0.05). In addition to ammonia production by DNRA, ammonia can be produced by proteolysis, and through metabolism of arginine and urea (Liu et al., 2012). Thus, lactate and ammonia may be linked by lactic acid producing bacteria (e.g., *Streptococcus*, *Lactobacillus* and *Actinomyces*) which can convert arginine into ammonia (Liu et al., 2012). Additionally, lactate can be used during, and stimulate, DNRA (van den Berg et al., 2017). We hypothesize that the salivary ammonia levels under the *in vivo* conditions of this study may partly reflect the amount of lactic acid producing bacteria, or that they were metabolically linked to lactate in a different way. In dental plaque, where the clearance effect of saliva will be limited, ammonia could accumulate locally with time as previously observed *in vitro* (Rosier et al., 2020a).

The acceptable daily intake of nitrate is currently at 3.7 mg/kg of body weight (EFSA, 2017). This limit is set because under certain conditions nitrate can form N-nitroso compounds, some of which cause cancer. This has been reported on processed meats to which nitrate salts are added as preservatives, resulting from bacterial reduction of nitrate to nitrite in meat and chemical reactions of nitrite with meat molecules (Skibsted, 2011; Sindelar and Milkowski, 2012). These chemical reactions are further stimulated in the acidic stomach. Another source of nitrate is drinking water and high nitrate levels in water, resulting from agricultural contamination, have been associated with cancer and other adverse health effects (Ward et al., 2018). However, we obtain over 80% of nitrate from vegetables, which are considered protective against cancer and other diseases (Link and Potter, 2004; Wang et al., 2014; Turati et al., 2015). This includes nitrate-rich vegetables such as lettuce and spinach, which are considered protective against different types of cancer (mouth, pharynx, larynx, oesophagus and stomach) (Mills et al., 2017). Therefore, nitrate outside of its natural context appears to be potentially harmful, whereas eating vegetables which provide the most nitrate, is beneficial for health and reduces the risk of cancer. Natural combinations of anti-oxidants and polyphenols in vegetables (and fruits) prevent N-nitroso compound formation and possibly damage (Chung et al., 2002; Ward, 2009; Kobayashi et al., 2015; Azqueta and Collins, 2016). Future assessments of nitrate ADI should take the source of nitrate into account.

Limitations of this work include the small number of participants for different experiments, the exclusion of caries active patients, the analysis of saliva as the only oral sample and monitoring only *Rothia* among nitrate-reducing bacteria by qPCR. Future studies should confirm these results in dental plaque samples of a larger group of participants, including caries active patients, ideally under fasting conditions to avoid the effect of dietary nitrate and other food components on physiological outcomes. Additionally, changes in other nitrate-reducing species (e.g., representatives of *Neisseria*) should be measured by qPCR for quantitative assessment. It should also be noted that the effect of nitrate was tested in combination with other potentially active molecules present in beetroot, such as polyphenols or antioxidants, and therefore nitrate from other vegetable sources or nitrate salts could have a different effect. Along with the acute protective effects of nitrate, long term nitrate intake should be studied as nitrate-reducing genera and enzymes may increase over time. The resilience against acidification when sugars are fermented could thus increase over time when nitrate-rich vegetables are consumed on a daily basis, and future studies should test this possibility.

## Concluding Remarks

To our knowledge, this is the first study to confirm a pH buffering effect of nitrate when sugars are fermented *in vivo*. The data demonstrate an acute effect measurable after 1-4 h of nitrate intake, and future studies should investigate the effect of daily nitrate exposure over longer periods of time. The main underlying mechanism appeared to be lactate usage by nitrate reducing bacteria, including *Rothia* and *Neisseria*. Thus, our data suggest that the amount of nitrate in nitrate rich-vegetables could act as an important anti-caries agent. In this respect, it is interesting to note that dental caries have been associated with diets low in fruits and vegetables (Moynihan and Petersen, 2004) – the food groups which naturally contain the most nitrate. In one study, children that ate fewer than five servings of fruit and vegetables per day had more caries in primary teeth (Dye et al., 2004). Additionally, in a study with 3689 Japanese children, the habit of eating vegetables before a meal was associated with a lower caries incidence in primary teeth (Ito et al., 2019). In addition to reducing the frequency of fermentable sugar intake and brushing twice per day with fluoridated toothpaste (Frencken et al., 2012), we hypothesize that increasing the amount of dietary nitrate could be an effective way to prevent caries development. The results in this study support the hypothesis that nitrate could be used as a prebiotic and certain nitrate-reducing bacterial strains as probiotics to prevent caries development. Furthermore, our results suggest that the addition of a nitrate source (e.g., vegetable extract or nitrate salt in combination with anti-oxidants) to oral hygiene products would be beneficial in this regard.

## Data Availability Statement

All measurements of this study can be found in the **Supplementary Spreadsheet**. The sequencing reads are deposited in the Sequence Read Archive under BioProject PRJNA725996.

## Ethics Statement

The studies involving human participants were reviewed and approved by Ethical Committee of DGSP-FISABIO (Valencian Health Authority). The patients/participants provided their written informed consent to participate in this study.

## Author Contributions

BR and AM contributed to the design of the work and drafted and revised the manuscript. BR and SG-E did the experimental work. CP determined the oral health status of the participants. BR, AA, AG, and CP contributed to data acquisition and analysis. All authors contributed to the article and approved the submitted version.

## Funding

AM was supported by a grant from the European Regional Development Fund and Spanish Ministry of Science, Innovation and Universities with the reference RTI2018-102032-B-I00, as well as a grant from the Valencian Innovation Agency with the reference INNVAL20/19/006. BR was supported by a FPI fellowship from the Spanish Ministry of Science, Innovation and Universities with the reference Bio2015-68711-R.

## Conflict of Interest

AM and BR are co-inventors in a pending patent application owned by the FISABIO Institute, which protects the use of nitrate as a prebiotic.

The remaining authors declare that the research was conducted in the absence of any commercial or financial relationships that could be construed as a potential conflict of interest.

## Supplementary Material

Supplementary Figure 1 Correlations between physiological parameters in all participants of this study at baseline and after supplement intake. In **(A–F)**, correlations between different physiological parameters are shown. The purple dots represent data at 1-4 h after nitrate-rich supplement intake (n = 24) and the grey dots at the baseline measurements on the same day (n=24). The black dotted lines are the linear regression curves when combining the baseline measurements with the measurements after supplement intake (total n = 48). The ΔpH is the salivary pH difference between the pre-sucrose measurement and the post-sucrose measurement (negative values indicate a pH drop). **(A)**: ΔpH and lactate detected post-sucrose. **(B)**: ΔpH and Δlactate. **C**: ΔpH and salivary nitrate (pre-sucrose) **(D)**: lactate detected post-sucrose and salivary nitrate (pre-sucrose). **(E)**: salivary ammonium (pre-sucrose) and lactate detected post-sucrose. **(F)**: ΔpH and Δammonium. Abbreviations: sup. = supplement, BL = baseline (0 h), comb. = combined, pre-sucr. = pre-sucrose measurements, post-sucr. = 10 min post-sucrose measurement. Spearman-Rho correlations (*r*) were calculated 1-4 h after supplement intake (sup.), at baseline (BL) or both combined (comb.). P-values and linear regression curves were shown if trends were found (p 0.05-0.1). *p < 0.05, ***p <0.001, ****p < 0.0001, n.s. = not significant. Δ = post-measurement – pre-measurement.

Supplementary Figure 2 Comparing the communities of 6 individuals at baseline (0 h) and 4 h after nitrate supplement or water intake. In **(A)**, bacterial composition data at the genus-level is shown and in **(B)**, at the species-level. Water 0 h and supplement 0 h are the baseline communities before intake of water or supplement, respectively. Water 4 h and supplement 4 h are the bacterial communities 4 h after intake of water or the supplement, respectively. The Adonis and CCA p-values between the different groups were not significant (p > 0.6). This suggest that the general community structures are not significantly different 4 h after nitrate intake compared to 4 h after water intake, although some differences in specific bacteria were detected (see **Figure 7** and **Supplementary Figure 4**).

Supplementary Figure 3 The effect of nitrate-rich supplements 1-4 h after intake on sucrose rinses compared to baseline measurements (n = 24). In this figure the 24 individuals from studies 1-3 are combined. The salivary pH **(A)** and concentrations of nitrate **(B)**, nitrite **(C)**, lactate **(D)** and ammonium **(E)** are shown. Sugar rinses were performed at baseline (BL, 0 h) and 1-4 h after intake of a nitrate-rich supplement. Saliva samples were collected right before the sugar rinse (pre-sucrose, white bars) and 10 min after the sugar rinse (post-sucrose, yellow bars). The bars and small white circles represent the averages and individual donors, respectively. Wilcoxon tests were used to compare the pre-sucrose with the post-sucrose measurements. Additionally, the BL pre- and post-sucrose measurement were compared with 1-4 h pre- and post-sucrose measurement, respectively. Significant changes (* p < 0.05, ** p < 0.01, *** p < 0.001, **** p < 0.0001) and trends (grey text, p = 0.05 - 0.1) were shown.

Supplementary Figure 4 Species level analysis of study 3 (comparing a nitrate rich supplement intake with water intake). In **(A)**, the species detected in the 6 individuals of study 3 are represented in bar charts. Based on the median, the 20 most abundant species are shown. The grey boxes with “other” contain species < 0.5% abundance grouped together. Changes in relative abundances of different species are shown in **(B)** (*Rothia dentocariosa*), **(C)** (*Rothia mucilaginosa*), **(D)** (*Prevotella salivae*) and **(E)** (*Corynebacterium durum*). The single dark cyan or red dots represent individual participants. Because of the small sample size, unadjusted p-values are shown.

Supplementary Table 1A Nitrate-rich beetroot supplement

Supplementary Table 1B Nitrate-poor placebo supplement
