# Supplementary figures and images for: A Single Dose of Nitrate Increases Resilience Against Acidification Derived From Sugar Fermentation by the Oral Microbiome
Source: Front Cell Infect Microbiol. 2021 Jun 3;11:692883. doi: 10.3389/fcimb.2021.692883 (PMC8238012; doi:10.3389/fcimb.2021.692883)

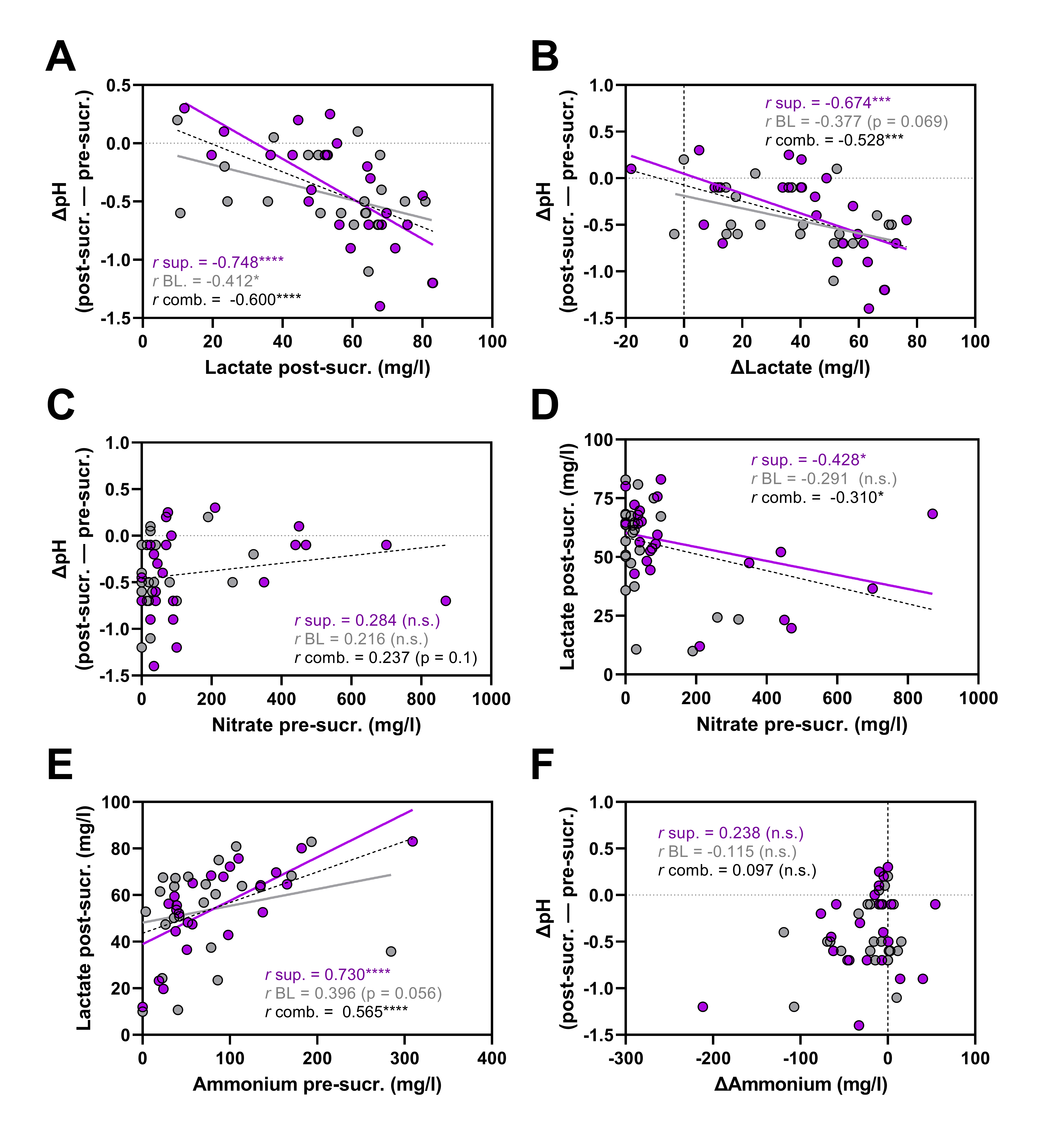

Supplement: Supplementary Figure 1 — Correlations between physiological parameters in all participants of this study at baseline and after supplement intake. In (A–F), correlations between different physiological parameters are shown. The purple dots represent data at 1-4 h after nitrate-rich supplement intake (n = 24) and the grey dots at the baseline measurements on the same day (n=24). The black dotted lines are the linear regression curves when combining the baseline measurements with the measurements after supplement intake (total n = 48). The ΔpH is the salivary pH difference between the pre-sucrose measurement and the post-sucrose measurement (negative values indicate a pH drop). (A): ΔpH and lactate detected post-sucrose. (B): ΔpH and Δlactate. C: ΔpH and salivary nitrate (pre-sucrose) (D): lactate detected post-sucrose and salivary nitrate (pre-sucrose). (E): salivary ammonium (pre-sucrose) and lactate detected post-sucrose. (F): ΔpH and Δammonium. Abbreviations: sup. = supplement, BL = baseline (0 h), comb. = combined, pre-sucr. = pre-sucrose measurements, post-sucr. = 10 min post-sucrose measurement. Spearman-Rho correlations (r) were calculated 1-4 h after supplement intake (sup.), at baseline (BL) or both combined (comb.). P-values and linear regression curves were shown if trends were found (p 0.05-0.1). *p < 0.05, ***p <0.001, ****p < 0.0001, n.s. = not significant. Δ = post-measurement – pre-measurement. [file Image_1.tif]

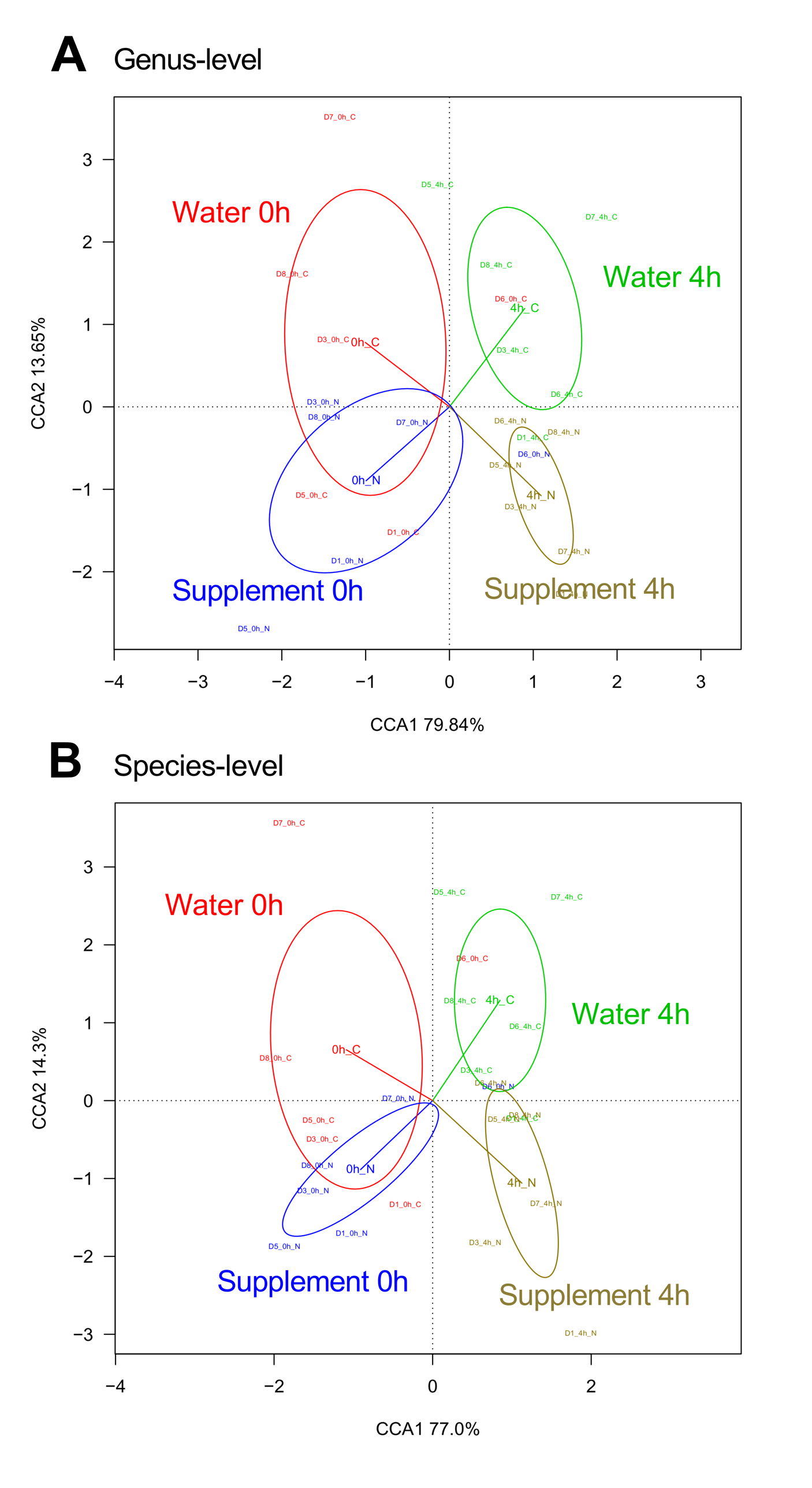

Supplement: Supplementary Figure 2 — Comparing the communities of 6 individuals at baseline (0 h) and 4 h after nitrate supplement or water intake. In (A), bacterial composition data at the genus-level is shown and in (B), at the species-level. Water 0 h and supplement 0 h are the baseline communities before intake of water or supplement, respectively. Water 4 h and supplement 4 h are the bacterial communities 4 h after intake of water or the supplement, respectively. The Adonis and CCA p-values between the different groups were not significant (p > 0.6). This suggest that the general community structures are not significantly different 4 h after nitrate intake compared to 4 h after water intake, although some differences in specific bacteria were detected (see Figure 7 and Supplementary Figure 4 ). [file Image_2.tif]
