# Supplementary material for: A Single Dose of Nitrate Increases Resilience Against Acidification Derived From Sugar Fermentation by the Oral Microbiome
Source: Front Cell Infect Microbiol. 2021 Jun 3;11:692883. doi: 10.3389/fcimb.2021.692883 (PMC8238012; doi:10.3389/fcimb.2021.692883)
Supplement: Supplementary Table 1A — Nitrate-rich beetroot supplement [file DataSheet_1.docx]

**Supplementary tables**

Supplementary Table 1a: nitrate-rich beetroot supplement

| **Ingredient** | **mg per dose of 11.5 g** |
| --- | --- |
| Beetroot extract 3% nitrate* (*Beta vulgaris*) | 8333 (= 250 mg nitrate)* |
| Apple Pectin E-440 | 2883 |
| Natural Red Fruits Flavor (Doler) | 200 |
| Vitamin C, L-Ascorbic Acid | 80 |
| Sucralose E-955 (richness 98% - 102%) | 4 |
| Ammonium molybdate (54.32% Mo) (Ammonium heptamolybdate) | 0.09205 (= 0.050 mg molybdenum) |
| Total | 11500 |

*HPLC measurement provided by manufacturer

Supplementary Table 1b: nitrate-poor placebo supplement

| **Ingredient** | **mg per dose of 11.5 g** |
| --- | --- |
| Orange powder (Doler) | 8333 (with <6 mg nitrate)* |
| Apple Pectin E-440 | 2883 |
| Natural Red Fruits Flavor (Doler) | 200 |
| Vitamin C, L-Ascorbic Acid | 80 |
| Sucralose E-955 (richness 98% - 102%) | 4 |
| Ammonium molybdate (54.32% Mo) (Ammonium heptamolybdate) | 0.09205 (= 0.050 mg molybdenum) |
| Total | 11500 |

**the analytical sensitivity of the colorimetric assay started at 6 mg, because of the orange color of the placebo*
